# Efficacy of a Personalized Mobile Health Intervention (BedTime) to Increase Sleep Duration Among Short-Sleeping Patients With Type 2 Diabetes: Protocol for a Pilot Randomized Controlled Trial

**DOI:** 10.2196/64023

**Published:** 2025-04-14

**Authors:** Yuki Ban, Kayo Waki, Ryohei Nakada, Akihiro Isogawa, Kengo Miyoshi, Hironori Waki, Shunsuke Kato, Hideaki Sawaki, Takashi Murata, Yushi Hirota, Shuichiro Saito, Seiji Nishikage, Atsuhito Tone, Mayumi Seno, Masao Toyoda, Shinichi Kajino, Kazuki Yokota, Yuya Tsurutani, Toshimasa Yamauchi, Masaomi Nangaku, Kazuhiko Ohe

**Affiliations:** 1 Professional Degree Program, School of Public Health, Graduate School of Medicine The University of Tokyo Tokyo Japan; 2 Department of Biomedical Informatics, Graduate School of Medicine The University of Tokyo Tokyo Japan; 3 Department of Diabetes and Metabolic Diseases, Graduate School of Medicine The University of Tokyo Tokyo Japan; 4 Division of Diabetes, Mitsui Memorial Hospital Tokyo Japan; 5 Department of Metabolism and Endocrinology, Akita University Graduate School of Medicine Akita Japan; 6 Center for Medical Education and Training, Akita University Hospital Akita Japan; 7 Sawaki Internal Medicine and Diabetes Clinic Osaka Japan; 8 Division of Diabetes and Endocrinology, Department of Internal Medicine Kobe University Graduate School of Medicine Kobe Japan; 9 Department of Internal Medicine, Diabetes Center Okayama Saiseikai General Hospital Okayama Japan; 10 Division of Nephrology, Endocrinology and Metabolism Department of Internal Medicine, Tokai University School of Medicine Isehara Japan; 11 Aikawa Comprehensive Internal Medicine Clinic Nagoya Japan; 12 Yokota Medical Clinic Akashi Japan; 13 Endocrinology and Diabetes Center, Yokohama Rosai Hospital Yokohama Japan; 14 Division of Nephrology and Endocrinology, Graduate School of Medicine The University of Tokyo Tokyo Japan

**Keywords:** digital therapeutics, behavior change, Theory of Planned Behavior, sleep duration, type 2 diabetes, randomized controlled trial

## Abstract

**Background:**

A strong association exists between sleep duration and glycemic control in patients with type 2 diabetes (T2D), yet convincing evidence of a causal link remains lacking. Improving sleep is increasingly emphasized in clinical T2D treatment guidance, highlighting the need for effective, scalable sleep interventions that can affordably serve large populations through mobile health (mHealth).

**Objective:**

This study aims to pilot an intervention that extends sleep duration by modifying bedtime behavior, assessing its efficacy among short-sleeping (≤6 hours per night) patients with T2D, and establishing robust evidence that extending sleep improves glycemic control.

**Methods:**

This randomized, single-blinded, multicenter study targets 70 patients with T2D from 9 institutions in Japan over a 12-week intervention period. The sleep extension intervention, BedTime, is developed using the Theory of Planned Behavior (TPB) and focuses on TPB’s constructs of perceived and actual behavioral control (ABC). The pilot intervention combines wearable actigraphy devices with SMS text messaging managed by human operators. Both the intervention and control groups will use an actigraphy device to record bedtime, sleep duration, and step count, while time in bed (TIB) will be assessed via sleep diaries. In addition, the intervention group will receive weekly bedtime goals, daily feedback on their bedtime performance relative to those goals, identify personal barriers to an earlier bedtime, and select strategies to overcome these barriers. The 12-week intervention period will be followed by a 12-week observational period to assess the sustainability of the intervention’s effects. The primary outcome is the between-group difference in the change in hemoglobin A_1c_ (HbA_1c_) at 12 weeks. Secondary outcomes include other health measures, sleep metrics (bedtime, TIB, sleep duration, total sleep time, and sleep quality), behavioral changes, and assessments of the intervention’s usability. The trial commenced on February 8, 2024, and is expected to conclude in February 2025.

**Results:**

Patient recruitment ended on August 29, 2024, with 70 participants enrolled. The intervention period concluded on December 6, 2024, and the observation period ended on February 26, 2025, with 70 participants completing the observation period. The data analysis is currently underway, and results are expected to be published in July 2025.

**Conclusions:**

This trial will provide important evidence on the causal link between increased sleep duration and improved glycemic control in short-sleeping patients with T2D. It will also evaluate the efficacy of our bedtime behavior change intervention in extending sleep duration, initially piloted with human operators, with the goal of future implementation via an mHealth smartphone app. If proven effective, this intervention could be a key step toward integrating sleep-focused mHealth into the standard treatment for patients with T2D in Japan.

**Trial Registration:**

Japan Registry of Clinical Trials jRCT1030230650; https://jrct.niph.go.jp/latest-detail/jRCT1030230650

**International Registered Report Identifier (IRRID):**

DERR1-10.2196/64023

## Introduction

Worldwide, approximately 537 million people aged 20-79 years have diabetes [1]. Diabetes is a serious public health concern, significantly impacting health care expenditures and human life, causing 4.2 million deaths annually [2,3]. Type 2 diabetes (T2D) is difficult to cure once it develops and, if left untreated, can lead to complications such as cardiovascular disease and microvascular damage [4].

T2D treatment guidelines include both pharmacological and lifestyle modification elements. Traditionally, lifestyle modifications have focused on diet and exercise, but recently, improving sleep in patients with T2D has gained prominence [5,6]. Some patients with T2D suffer from sleep disorders, including obstructive sleep apnea, restless leg syndrome, and insomnia, for which treating the underlying disorder is the appropriate approach [7]. Sleep quality is associated with T2D risk [8], and interventions to improve sleep quality have shown effectiveness [9]. Many patients with T2D do not get sufficient sleep, experiencing what is termed *short sleep*. Definitions of short sleep vary across studies, ranging from 6 or fewer hours per night to less than 4.5 hours per night [10]. This issue is widespread, with data indicating that 39.4% of patients with T2D in Japan sleep fewer than 6.4 hours per night [11].

There is strong evidence that short sleep is associated with an increased risk of T2D and poorer glycemic control. In Japanese patients with T2D, short sleep is linked to higher hemoglobin A_1c_ (HbA_1c_) levels [11], with a meta-analysis indicating a 0.23% difference in HbA_1c_ due to short sleep [12]. Prospective studies have also shown an increased risk of T2D associated with short sleep [8]. Plausible biological mechanisms support these findings, as sleep extension has been shown to reduce energy intake [13], while sleep restriction worsens insulin sensitivity [14].

Surprisingly, there is a lack of strong evidence for causality—specifically, increasing sleep duration in short-sleeping patients with T2D without sleep disorders leads to improvements in HbA_1c_ levels. Recent meta-analyses [15,16] found limited and conflicting evidence on the effect of sleep interventions on glycemic control. Some studies have investigated interventions for patients with T2D with insomnia [17,18]. One study examined an educational intervention for patients with T2D, including those with short sleep, but results from a published abstract [19] are unclear, and no full paper has been published. Two related studies used cognitive behavioral therapy to improve HbA_1c_ by enhancing sleep quality rather than duration [20,21]. Overall, strong evidence is lacking to support the idea that increasing sleep duration in short-sleeping patients with T2D without sleep disorders leads to improved HbA_1c_ levels.

Numerous studies have successfully increased sleep duration in various populations. A recent meta-analysis of 42 studies found that extending time in bed (TIB) has the greatest potential to modify sleep, with direct interventions (ie, scheduling a longer period in bed) resulting in an average increase of 1 hour and 23 minutes in sleep duration [22].

Mobile health (mHealth) interventions, such as mobile phone apps supporting self-management, have been shown to be effective in increasing physical activity, reducing body weight [23,24], and improving glycemic control [25,26]. However, to our knowledge, no mHealth apps have been designed specifically to improve sleep duration in short-sleeping patients with T2D without sleep disorders. Personalization of intervention components has been shown to enhance patient motivation and engagement with the app [27]. Several studies have found that tailored interventions are more effective at changing health behaviors than nontailored ones [28,29]. Moreover, interventions based on behavior change theory have been shown to be more effective than those lacking a theoretical foundation [30], with those based on the Theory of Planned Behavior (TPB) being particularly effective [31]. TPB emphasizes intention and ABC as key determinants of behavior, with perceived behavioral control (PBC)—similar or identical to Bandura’s self-efficacy—playing a central role in shaping intention [32,33].

To address the lack of evidence on whether improvements in sleep duration lead to better glycemic control, we developed a pilot study of a personalized mHealth intervention, BedTime, designed to increase sleep duration and assess its impact on glycemic control in short-sleeping patients with T2D. The intervention is based on the TPB framework to promote behavior change toward earlier bedtimes. This trial aims to evaluate the efficacy of the BedTime approach in a 12-week, single-blind, randomized controlled pilot trial. The specific research objectives and hypotheses are as follows:

- Objective 1: To evaluate the efficacy of the BedTime approach in shifting patients to an earlier bedtime, as measured by daily bedtime reporting, and increasing sleep duration, as measured by daily actigraphy.
- We hypothesize that, by the end of the intervention, the intervention group will show statistically significant changes in bedtime and sleep duration compared with the control group.
- Objective 2: To evaluate the efficacy of the BedTime approach in reducing HbA_1c_ levels.
- We hypothesize that, by the end of the intervention, the intervention group will show a statistically significant reduction in HbA_1c_ levels compared with the control group.

## Methods

### Study Overview

**Figure 1:**
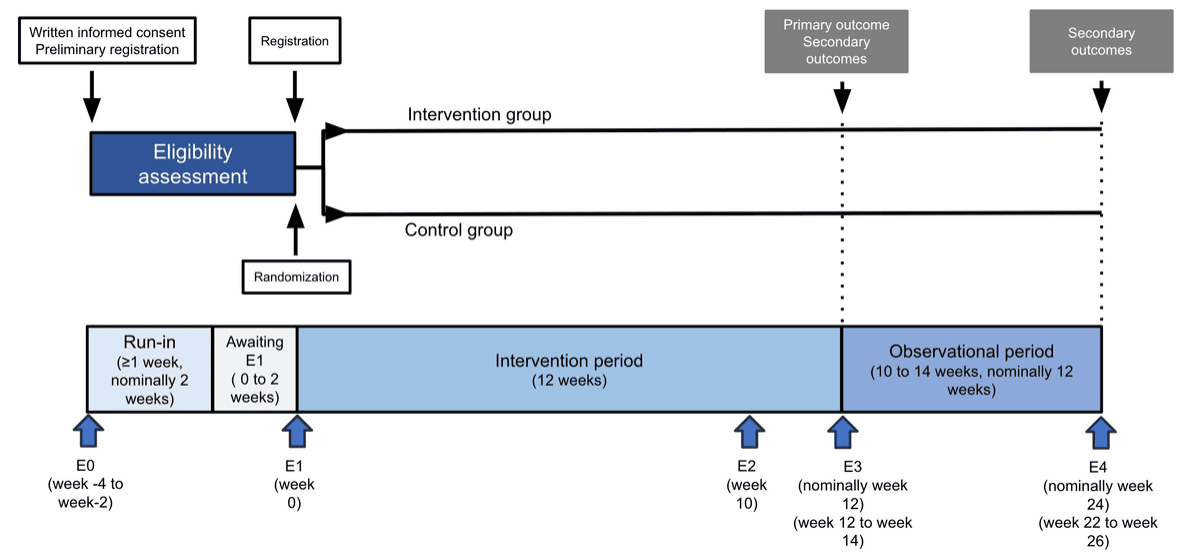
Overview of the study timeline.

This single-blinded, multicenter pilot study is a 2-arm randomized controlled trial designed to evaluate the efficacy of the BedTime mHealth intervention in promoting earlier bedtime behavior and increasing sleep duration among short-sleeping patients with T2D. The trial will be conducted and reported in accordance with CONSORT (Consolidated Standards of Reporting Trails) guidelines. As outlined in Figure 1, the study begins with recruitment (E0), followed by a baseline measurement period (nominally 2 weeks, but at least 7 days), and then randomized assignment to intervention and control groups (E1). Patients in the intervention group will record their daily bedtime, sleep duration, and step count using an actigraphy device, with TIB assessed via sleep diaries. They will receive weekly bedtime goals, daily feedback on their bedtime performance relative to these goals, and guidance to identify personal barriers to an earlier bedtime along with strategies to overcome them. The control group will also record daily bedtime, sleep duration, and step count using an actigraphy device, with TIB assessed via sleep diaries, but they will receive no feedback and will not be assigned goals. During week 10 of the intervention period (E2), the intervention group will complete a TPB questionnaire. The intervention will last for 12 weeks, followed by end-of-intervention assessments of the primary and secondary outcomes (E3). After the intervention period, there will be an observation phase, nominally 12 weeks in length, but its duration will depend on the date of the final visit (E4), which may occur between 10 and 14 weeks after the intervention ends. The study will be conducted over 1 year, from February 8, 2024, to January 22, 2025.

### Intervention Design

Some features of the intervention are shared by both the control and intervention groups, while others are unique to the intervention group (Table 1). This mHealth intervention incorporates a physical activity monitoring system (iAide2; Tokai) and a researcher-designed paper diary for patients in both groups. The iAide2 measures activity intensity levels, skin temperature, and pulse rate [34,35] to assess step count and sleep, providing minute-by-minute wake/sleep assessments [36,37]. Additionally, the iAide2 enables timely behavioral feedback via a wirelessly connected Event button and near-real-time data access for researchers through an administrative screen. During the 12-week intervention period, patients in both the control and intervention groups wear the iAide2 device at all times except when bathing and press its Event button at bedtime each day. Patients record their bedtime and risetime daily in the paper diary. For the intervention group, the diary includes additional pages implementing specific aspects of the intervention. Bedtime is defined as the time when a patient begins trying to sleep, and risetime as the time when they stop trying to sleep. Diary entries are used to calculate TIB, the duration between bedtime and risetime. The intervention is designed to encourage an earlier bedtime, with the expectation that this will extend TIB and thereby increase sleep duration (ie, the portion of the TIB interval during which the patient is asleep).

**Table 1:** Intervention features for the intervention and control groups.

Features			Intervention group		Control group
**iAide2**					
	Press the Event button to send bedtime	Yes		Yes	
	Measure daily sleep and steps (without providing this information to patients)	Yes		Yes	
**Diary**					
	Enter bedtime and risetime	Yes		Yes	
	Define barriers and coping strategies and report the number of coping strategies implemented	Yes		No	
	TPB^a^ questionnaire at E2	Yes		No	
**SMS text message**					
	Diary reminder	Yes		Yes	
	Low battery message	Yes		Yes	
	Daily feedback and target bedtime	Yes		No	
	Request for a reply message when patients forget to press the Event button	Yes		No	
**Surveys and questionnaires**					
	Screening at E0	Yes		Yes	
	Sleep and Transtheoretical Model Questionnaire at E0, E3, and E4	Yes		Yes	
	Feedback survey	Yes		No	
	TPB questionnaires at E1	Yes		No	
	Ask if taking any medication that may affect one’s sleep at E0, E1, E3, and E4	Yes		Yes	
	Obtain information about medications, including type 2 diabetes treatments and other drugs, either by asking the patients or by checking their medical records at E0, E1, E3, and E4	Yes		Yes	
	Measure height at E0, and measure weight and blood pressure at E0, E1, E3, and E4	Yes		Yes	
	Ask about any adverse events or malfunction in iAide2 at E1, E3, and E4	Yes		Yes	
	TPB questionnaires at E3 and E4	Yes		Yes	

^a^TPB: Theory of Planned Behavior.

**Figure 2:**
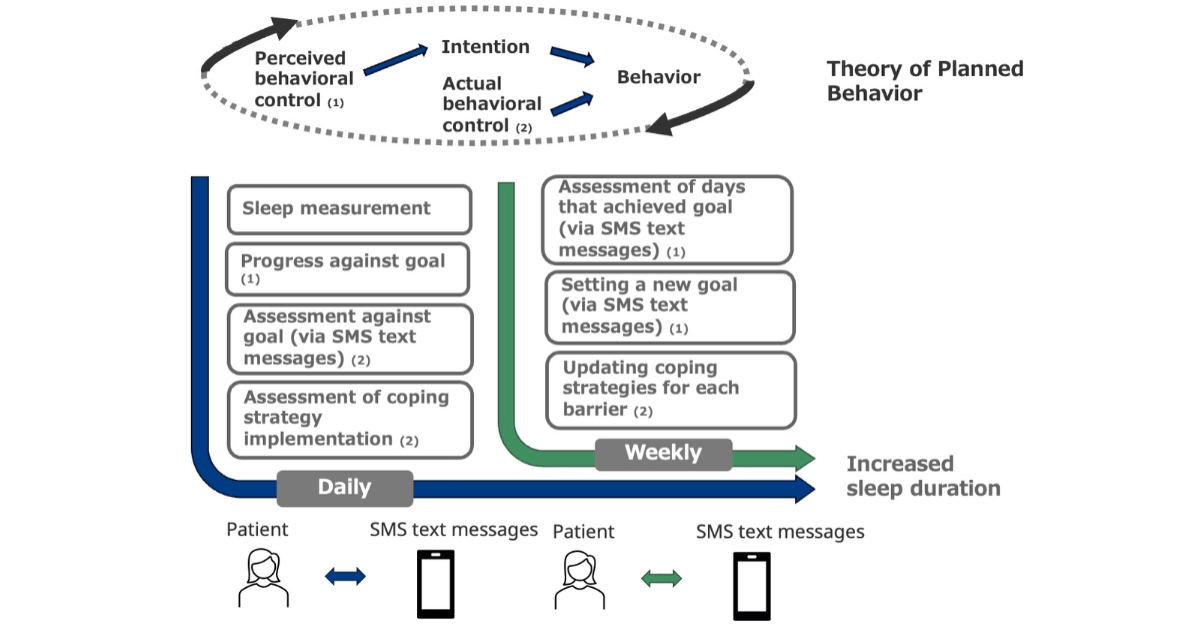
BedTime’s process for implementing Theory of Planned Behavior–based personalization.

For the intervention group, daily and weekly messaging will deliver a TPB-based intervention targeting PBC and ABC (Figure 2). A researcher or clinical research coordinator (CRC), under the supervision of the researcher, will establish a baseline bedtime and set an initial goal for an earlier bedtime. They will also assist patients in identifying barriers to an earlier bedtime and developing coping strategies. Intervention group patients will receive a daily SMS text message assessment of their performance relative to their target bedtime and will record whether they implemented their coping strategies each day. They will also receive weekly assessments of their progress toward the goal, along with an updated target bedtime, via weekly SMS text messages. Additionally, they will determine coping strategies for the upcoming week.

**Figure 3:**
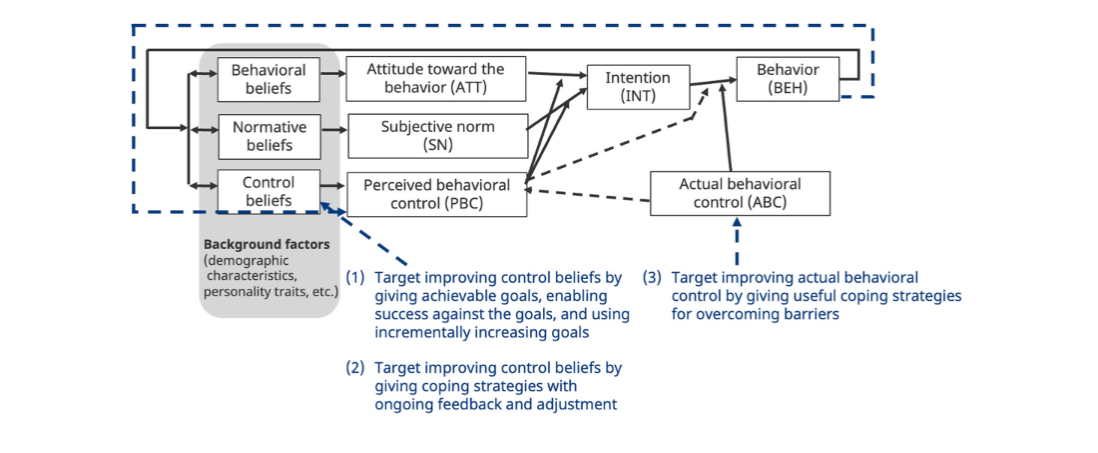
Theory of Planned Behavior applied to the BedTime intervention.

The intervention is based on the TPB framework (Figure 3) [32,33]. According to TPB, behavior is driven by an individual’s intention to perform the behavior and their ABC, or ability to do so. A behavior is more likely to occur when both intention and actual ability are high. Intention itself is influenced by 3 factors: personal attitude toward the behavior, subjective norms or the perceived expectations of important others, and PBC, which reflects one’s confidence in their ability to perform the behavior. Each of these 3 drivers of intention (attitude toward the behavior, subjective norms, and PBC) is, in turn, influenced by the person’s beliefs about them. The stronger the positive behavioral, normative, and control beliefs, the more likely an individual is to intend to perform the behavior. Actually carrying out the behavior depends on both intention and ABC. As ABC is difficult to measure, researchers generally use PBC as a proxy for ABC in predicting behavior [32,38].

The BedTime intervention is designed to influence PBC by modifying control beliefs. The primary strategy for improving control beliefs—and thus PBC—is to set achievable goals, enabling patients to experience success and build positive self-mastery. We implement progressively challenging but attainable goals using a simple algorithm based on our prior work [39]. This algorithm adjusts the suggested target bedtime each week based on the number of days the user met their goal in the previous week. Adjustments include increases of 5, 10, or 15 minutes and decreases of 5 or 10 minutes, depending on past target bedtime achievement levels.

The secondary mechanism for improving control beliefs involves daily self-reflection on barriers and coping strategies, reinforcing patients’ sense of control over their behavior. These mechanisms align with those used to enhance self-efficacy in social cognitive theory [32]. Beyond influencing PBC, the barrier and coping strategy intervention may also improve ABC by providing useful coping strategies. However, its impact on ABC is expected to be minor, as the suggested strategies may not be entirely novel or groundbreaking for patients. Each week, participants select or write down 1-3 barriers to achieving their target bedtime and propose a coping strategy for each (Table 2). Participants can select coping strategies from a literature-based list of solutions to common barriers to early bedtime [40-60] or describe their own. At the end of each day, they use the diary to report the number of coping strategies they implemented.

**Table 2:** Example of personalized coping planning strategy (with English translation).

Japanese		English translation	
Barriers	Coping strategies	Barriers	Coping strategies
遅い時間に夕食を食べて、まだやるべきことがある	就寝時刻の少なくとも2時間前に夕食を終える	I eat dinner late and still have things to do.	Finish dinner at least 2 hours before bedtime.
遅い時間にお風呂に入って、まだやるべきことがある	寝る1～2時間前にぬるめのお風呂に入る	I take a bath late and still have things to do.	Take a lukewarm bath 1-2 hours before going to bed.
就寝前にやるべき仕事がある	もっと睡眠をとるようにという医師の指示を上司に伝え、終業時間を早めるよう交渉する	I have work to do before bed	Tell your boss about your doctor’s orders to get more sleep and negotiate an earlier finish time.
仕事から帰ってくるのが遅いため、まだやるべきことがある	もっと睡眠をとるようにという医師の指示を上司に伝え、終業時間を早めるよう交渉する	I’m late coming home from work, so I still have things to do.	Tell your boss about your doctor’s orders to get more sleep and negotiate an earlier finish time.
家事など、まだ手をつけていないことがある	就寝1時間前を空けるようにスケジュールを組み立てる	There are things I haven’t gotten around to yet, such as housework.	Plan your schedule so that you leave an hour before bedtime.
N/A^a^	やることのリストを作成し、就寝時ではなく翌日にそれをすることに専念する	N/A	Make a to-do list and commit to doing it the next day instead of at bedtime.
N/A	より多くの睡眠をとるようにという医師の指示に基づいて、他の人の助けを借りて家事をする	N/A	Do housework with the help of others based on your doctor’s instructions to get more sleep
就寝時に高血糖または低血糖を管理する必要がある	必要な食事療法や投薬治療が就寝時刻までに完了するように、就寝時刻の 30 分～60 分前に血糖値を測定する	I need to manage hyperglycemia or hypoglycemia at bedtime	Measure your blood sugar 30-60 minutes before bedtime to ensure that any necessary dietary or medication therapy is completed by bedtime.
もう少し「自分」の時間が必要だ	「自分」の時間として、日中に 10 分間の休憩をいくつか取り入れる	I need a little more “me” time.	Incorporate several 10-minute breaks during the day for “me” time.

^a^N/A: not applicable.

The intervention provides daily individualized feedback via an SMS text message (Table 3). These messages, typically sent between 12 PM and 6 PM, inform the patient whether they went to bed earlier or later than their target bedtime the previous night, using an encouraging tone. This timing ensures that participants receive feedback well before their usual bedtime, allowing them sufficient time to adjust their nightly activities accordingly. The exact delivery time of the SMS text messages varies slightly due to the manual nature of the sending process.

At the end of each week, the intervention provides feedback via an SMS text message reporting the number of days the goal was achieved.

**Table 3:** Example of daily feedback (with English translation).

Situation	SMS text message	English translation
Last night, bedtime was earlier than the target bedtime which is 10 minutes earlier than the baseline bedtime.	昨夜は、「少なくとも 10分早く就寝する」という目標を達成できました。よく頑張りました! 健康を改善するための行動ができています！	Last night you achieved your goal of going to bed at least 10 minutes earlier. Great job! You’re taking action to improve your health!
Last night, bedtime was 15 minutes later than the goal bedtime of 30 minutes earlier than the baseline bedtime.	昨夜は15分遅く就寝しましたね。「30分早く就寝する」という目標には達しませんでしたが、今夜は目標就寝時刻の達成に向けてがんばりましょう。 あなたならできます！ お選びになった対処方法を確認して、今夜の就寝時刻を考えてください。 早く寝ると健康増進につながります！	You went to bed 15 minutes later than your goal last night. You didn’t reach your goal of going to bed 30 minutes earlier than your baseline bedtime. Try your best to reach your target bedtime tonight. You can do it! Review your chosen coping strategy and think about your bedtime tonight. Sleeping early will improve your health!

### Patient Recruitment

Patients will be recruited from 9 medical institutions in Japan (Textbox 1). Attending physicians will conduct recruitment during patients’ regular outpatient consultations. The recruitment period will run from February 8, 2024, to July 30, 2024.

**Participating institutions: 11 medical centers in Japan.**

- The University of Tokyo Hospital
- Mitsui Memorial Hospital
- Meiji Yasuda Life Insurance Company Tokyo Hospital
- Kobe University Hospital
- Akita University Hospital
- Tokai University Hospital
- Okayama Saiseikai General Hospital
- Sawaki Internal Medicine and Diabetes Clinic
- Yokota Medical Clinic
- Aikawa Comprehensive Internal Medicine Clinic
- Yokohama Rosai Hospital

To detect a 0.3% difference in the primary outcome (change in HbA_1c_), assuming an SD of 0.41% (the average observed in 2 prior studies [17,61]), and to achieve a 2-sided significance level of .05 with 80% statistical power, the minimum required sample size is 31 patients per group. Considering an estimated dropout rate of approximately 10%, we aim to recruit a total of 70 patients (35 in the intervention group and 35 in the control group).

All participants will receive a comprehensive written and verbal explanation before providing written informed consent. Eligibility screening will be conducted only after consent is obtained. If any findings related to efficacy or safety arise during the trial that may impact patient consent, we will promptly disclose this information to participants and obtain renewed consent.

We will use survey instruments as part of the inclusion and exclusion criteria (Textbox 2). To recruit patients who are both likely to benefit and capable of participating, we will include only those meeting all inclusion criteria (Textbox 3) while ensuring they do not meet any exclusion criteria (Textbox 4), following the methods of a prior study [62]. We anticipate a roughly equal sex distribution and an average participant age in the late 50s. Women undergoing menopausal transition will be included if they meet the study’s criteria.

We will use a Transtheoretical Model (TTM) questionnaire to categorize participants into the contemplation stage (willing to change health behavior within the next 6 months), preparation stage (willing to change health behavior within the next month), or action stage (has already made modifications to health behavior) [63]. In our previous study, patients in the contemplation and preparation stages were more likely to prefer smartphone-based self-management tools than those in the precontemplation stage [64]. Therefore, we will target patients in the contemplation, preparation, or action stage of increasing their sleep duration by 1 hour per day.

**Screening surveys.**

- STOP-J (Snoring, Tiredness, Observed Apnea, High Blood Pressure Screen, Japanese version): screening for obstructive sleep apnea [65]
- ISI-J (Insomnia Severity Index, Japanese version): screening for insomnia [66]
- IRLSQ-J version 2.2 (International Restless Legs Syndrome Study Group Rating Scale, Japanese version): screening for restless legs syndrome [67]
- AUDIT-C-J (Alcohol Use Disorders Identification Test—Consumption, Japanese version): screening for alcohol use disorder [68,69]
- PHQ-8-J (Patient Health Questionnaire-8, Japanese version): screening for major depression [70,71]
- TTM (Transtheoretical Model) Sleep Questionnaire: assess the TTM stage [63] relative to sleeping 7 or more hours nightly

**Study inclusion criteria (selecting patients who are likely to benefit from the intervention and are likely to be capable of participating).**

At the time of consent acquisition (E0)

- Diagnosed with type 2 diabetes and attending the hospital/clinic
- Hemoglobin A_1c_ (HbA_1c_) is 7.5% or higher at the time of obtaining consent
- A patient with short sleep (self-reports an average sleep duration in the prior month of 6 or fewer hours)
- In the contemplation, preparation, or action stage of the Transtheoretical Model (TTM) for the action of sleeping 7 hours or more per day
- Willing and able to wear a wristband-type measuring device on the nondominant wrist during the research period
- Has and uses a mobile phone capable of sending and receiving SMS text messages
- Able to attend consultations at designated times during the research period
- 18 years or older
- Fluent in spoken and written Japanese
- Does not have impaired cognitive function as determined by the investigator or subinvestigator
- Can undergo sleep treatment, as determined by the investigator or subinvestigator
- Fully informed about participation in this study, and has given free and voluntary written consent based on a thorough understanding of the study

**Exclusion criteria, focusing on patients who may not be able to participate safely or whose participation may interfere with the effectiveness of the study.**

1. Exclusion criteria at the time of consent acquisition (E0)
  - BMI is greater than 35.0 kg/m^2^
  - Age is 76 years or older
  - A shift worker or someone who occasionally deviates from a single main sleep period at night
  - A caregiver (of a child or adult) who needs to wake up during the night
  - At high risk of obstructive sleep apnea (STOP-J [Snoring, Tiredness, Observed Apnea, High Blood Pressure Screen, Japanese version] score of 3 or higher)
  - At high risk of insomnia (ISI [Insomnia Severity Index, Japanese version] score of 15 or higher)
  - Possibility of restless legs syndrome (IRLSQ-J [International Restless Legs Syndrome Study Group Rating Scale, Japanese version] score of 1 or higher)
  - High possibility of depression (PHQ-8 [8-item Patient Health Questionnaire] score of 10 or higher)
  - Taking medications that affect sleep
  - Changed type 2 diabetes medication within the past 8 weeks
  - Currently participating in another clinical research program
  - A heavy user of alcohol (AUDIT-C-J [Alcohol Use Disorders Identification Test—Consumption, Japanese version] score of 4 or higher for females, and 6 or higher for males)
  - Has severe chronic pain (due to cancer, etc)
  - Has a diagnosed clinical sleep disorder
  - Has an uncontrolled psychiatric disorder
  - Pregnant at the time of consent acquisition
  - Any other reason why the patient is classified as unfit for participation by the investigator or subinvestigator
2. Exclusion criteria before allocation (E1)
  - Recorded both bedtime and risetime in a diary for fewer than 7 days during the trial period
  - Recorded the Event button of the iAide2 fewer than 7 days during the trial period
  - Recorded fewer than 7 days of valid iAide2 data showing sleep duration, as judged by the investigator or subinvestigator

We will provisionally register patients who meet the eligibility criteria at E0. At this stage, each participant will receive an actigraph (iAide2) and will be instructed to wear it 24 hours a day, except when bathing, to press the Event button at bedtime, and to record their bedtimes and risetimes in the diary. Patients who fail to press the Event button for 7 or more days or fail to complete the diary for 7 or more days during the run-in period will be excluded to ensure adequate participation. Data collected during the run-in period will be used to confirm eligibility and establish baseline sleep and step counts. We will randomize eligible participants in a 1:1 ratio to either the intervention or control group using covariate-adaptive randomization by minimization to ensure balance across age (<65 and ≥65 years), sex, HbA_1c_ (<8.5% and ≥8.5%), and institution [72]. Random allocation will be conducted via the Internet Data and Information Center for Medical Research cloud, provided by the University Hospital Medical Information Network, an internet-based central randomization system. A research co-investigator will access the University Hospital Medical Information Network Internet Data and Information Center for Medical Research cloud and input the required baseline information. The system will then securely generate the allocation result immediately before assigning each participant to a group. This process ensures that researchers cannot access or predict allocation results in advance, minimizing potential bias.

### Intervention Process

The study (Table 4) consists of 5 events (E0-E4), a 2-week run-in period, a 12-week intervention period, and a 12-week observation period. Initial measurements will be taken at event E0. Questionnaires will be used to assess sleep behavior and gather patient feedback (Textbox 5).

Following randomization, the intervention begins. Before explaining the intervention, the researcher or CRC will administer the TPB Sleep Questionnaire and the Sleep-Related Behavior (SRB) Questionnaire to the intervention group. The researcher or CRC will then provide a diary customized for the intervention group. To establish a baseline bedtime, the researcher or CRC will average bedtimes recorded during the run-in period, considering any exclusions due to unusual circumstances in consultation with the patient. Patients will be informed that their ultimate goal is to shift their bedtime 1 hour earlier than the baseline, with an initial target of going to bed 10 minutes earlier during the first week. The researcher or CRC will help patients identify barriers preventing them from going to bed early and assist them in selecting up to 3 coping strategies tailored to these barriers for the first week. During this process, the researcher or CRC will facilitate discussions using the list in the diary as a reference. Patients may either choose strategies from the list or write their own in the diary. For the control group, the researcher or CRC will provide a diary similar to the one used during the run-in period but covering 12 weeks. Control group patients will be instructed to continue recording their bedtime and risetime as they did during the run-in period. The intervention period lasts 12 weeks, from E1 to E3. We will send reminder SMS text messages to both groups at the end of weeks 1 and 10 to encourage diary use. All equipment, including the diary, will be collected at E3 before the follow-up observation period begins.

For the intervention group, we will provide both daily and weekly feedback. Researchers will monitor the receipt of the Event time, and if it is missing, they will send a reminder asking the patient to report their bedtime via an SMS text message. If no bedtime is reported through either the Event record or SMS text message, the night will be considered a failure to achieve the target bedtime. At E2, 10 weeks after E1, we will send a reminder for participants to complete the TPB Sleep Questionnaire in the diary.

During the study, we will not restrict the use of supplements or drinks that may affect blood glucose and blood pressure. In both the intervention and control groups, T2D treatment—including oral T2D medications, insulin, glucagon-like peptide 1 receptor agonists, and medication dosage—may be adjusted at the discretion of the attending physician. We will collect information on T2D-related medication and medications affecting sleep at E1, E3, and E4.

Discontinuation is defined as a patient expressing a decision to withdraw or failing to record data for a continuous period of 7 or more days. If a participant discontinues during the intervention period, we will attempt to collect E3 measurement items. If discontinuation occurs during the observation period, we will attempt to collect E4 measurement items.

**Table 4:** Study events—1 measurement period and 4 visits spanning approximately 26 weeks.

Event	Focus	Time	Key activities
E0	Physical baseline assessments and initial registration	Enrollment: ≥7 days before the beginning of the intervention	Collect consent Collect demographic information: age, sex, date of type 2 diabetes diagnosis, smoking and drinking habits, macrovascular disease, diabetic retinopathy, periodontal disease, tinea pedis, diabetic neuropathy, and other medical history Collect physical data: hemoglobin levels, albumin, uric acid, blood urea nitrogen, estimated glomerular filtration rate, creatinine, fasting blood glucose, HbA_1c_^a^, low-density lipoprotein cholesterol, high-density lipoprotein cholesterol, triglycerides, urine specific gravity, urinary protein, urine albumin-to-creatinine ratio, height, body weight, and blood pressure measurements Collect medication status Conduct screening surveys (STOP-J^b^, ISI-J^c^, IRLSQ-J version 2.2^d^, AUDIT-C-J^e^, PHQ-8-J^f^, and TTM^g^ Sleep Questionnaire) Conduct PSQI-J^h^ survey Distribute actigraph (iAide2) and run-in period diary
Run-in	Baseline	From E0 for 2 weeks	Patients record daily bedtime and risetime, with automated (blinded) measurements of sleep parameters and step count
E1	Registration, allocation, and beginning of the intervention	Beginning of the intervention	Verify that run-in diary and iAide recording do not cause exclusion Finalize registration and allocation Collect medication status Intervention only: conduct surveys (TPB^i^ Questionnaire, SRB^j^ Questionnaire) Distribute the intervention diary or the control group diary Check for malfunctions or adverse events during run-in
Intervention period	Ongoing measurement	From E1 for 12 weeks	Patients record daily bedtime and risetime, with automated (blinded) measurements of sleep parameters and step count Intervention only: daily feedback, weekly self-set barriers and coping strategies via diary, weekly personalized target bedtime setting via SMS text messages
E2	Ongoing measurement	End of 10 weeks from E1	Intervention only: conduct TPB Questionnaire via diary
E3	End of the intervention	End of 12 weeks from E1	Collect physical data: hemoglobin levels, albumin, uric acid, blood urea nitrogen, estimated glomerular filtration rate, creatinine, fasting blood glucose, HbA_1c_, low-density lipoprotein cholesterol, high-density lipoprotein cholesterol, triglycerides, urine specific gravity, urinary protein, urine albumin-to-creatinine ratio, body weight, and blood pressure measurements Collect medication status Conduct surveys (TTM Sleep Questionnaire, TPB Questionnaire, SRB Questionnaire, PSQI-J) Intervention group: conduct survey (feedback survey) Collect actigraph (iAide2) and intervention period diary Check for malfunctions or adverse events
E4	End of follow-up	End of 24 weeks from E1	Collect physical data: hemoglobin levels, albumin, uric acid, blood urea nitrogen, estimated glomerular filtration rate, creatinine, fasting blood glucose, HbA_1c_, low-density lipoprotein cholesterol, high-density lipoprotein cholesterol, triglycerides, urine specific gravity, urinary protein, urine albumin-to-creatinine ratio, body weight, and blood pressure measurements Collect medication status Conduct surveys (TTM Sleep Questionnaire, TPB Questionnaire, SRB Questionnaire, PSQI-J)

^a^HbA_1c_: hemoglobin A_1c_.

^b^STOP-J: Snoring, Tiredness, Observed Apnea, High Blood Pressure Screen, Japanese version.

^c^ISI-J: Insomnia Severity Index, Japanese version.

^d^IRLSQ-J version 2.2: International Restless Legs Syndrome Study Group Rating Scale, Japanese version.

^e^AUDIT-C-J: Alcohol Use Disorders Identification Test—Consumption, Japanese version.

^f^PHQ-8-J: Patient Health Questionnaire-8, Japanese version.

^g^TTM: Transtheoretical Model.

^h^PSQI-J: Pittsburgh Sleep Quality Index, Japanese version.

^i^TPB: Theory of Planned Behavior

^j^SRB: Sleep-Related Behavior.

**Assessment questionnaires and surveys.**

- TPB (Theory of Planned Behavior) Sleep Questionnaire: assess individual components of TPB [32,33] relative to implementing an earlier bedtime
- SRB (Sleep-Related Behavior) Questionnaire: self-assessment of sleep over the past 2 weeks
- PSQI-J (Pittsburgh Sleep Quality Index, Japanese version): assess sleep quantity and quality [73,74]
- Feedback survey: collect feedback on the intervention

### Monitoring, Quality Control, and Data Management

An auditor, independent of the departments involved in the trial, including those responsible for monitoring, will inspect the medical institution and other facilities to ensure the trial is conducted appropriately.

### Ethical Approval

This trial will be conducted in compliance with the Declaration of Helsinki, the Pharmaceutical and Medical Device Act, the Ministerial Ordinance on Good Clinical Practice for Medical Devices, and all other relevant guidelines (jRCT1030230650). The study was approved by the Institutional Review Board of the University of Tokyo School of Medicine (approval number 2023336NI). We will obtain written informed consent from all study participants, and all patient data will be pseudonymized. Patients will receive compensation of JPY 30,000 (approximately US $200) at the end of E4.

The participant information materials and informed consent form are available from the corresponding author (KW) upon request. Any amendments to the protocol will be submitted for review and approval by the institutional review board. Study findings will be disseminated through peer-reviewed publications and conference presentations.

### Outcome Measures

#### Primary Outcome

The primary outcome of this study is the between-group difference in the change in HbA_1c_ from baseline (E0) to either the end of the intervention period at week 12 (E3) or, for patients who discontinue before E3, the point of discontinuation. A reduction of 0.3 percentage points in HbA_1c_ is considered clinically significant [75,76].

#### Secondary Outcomes

Secondary outcomes (Table 5) include health measures, bedtime and sleep measurements, other behavior change assessments, and evaluations of actigraph and diary usage. We will also assess safety outcomes, including hypertension requiring medical assistance (evaluated from system records), subjective hypoglycemia, lower back pain, and pain in the lower extremities (tarsus, thighs, knees, calves, shins, ankles, and feet), as well as any other adverse events (all assessed through patient interviews).

**Table 5:** Secondary outcomes investigated in the study.

Measurements	Outcomes
Differences in changes	Hemoglobin A_1c_ (%) (only after the end of the observation period)^a^ Weight (kg)^a^ BMI (kg/m^2^)^a^ Systolic blood pressure/diastolic blood pressure (mmHg)^a^ Estimated glomerular filtration rate (mL/min/1.73 m2)^a^ Fasting blood sugar (mg/dL)^a^ Low-density lipoprotein cholesterol (mg/dL)^a^ High-density lipoprotein cholesterol (mg/dL)^a^ Neutral fat (mg/dL)^a^ Urine albumin-to-creatinine ratio (mg/gCr)^a^ PSQI-J^a,^^b^
Differences in the measured values	Survey on past bedtimes^a^
Difference in proportions	Behavior change questionnaire^a^
Observed data	Elements of the bedtime questionnaire: attitude, subjective norms, perceived behavioral control, intention^c^ Sleep time calculated from iAide2’s sleep data and diary data^d^ Time in bed calculated from bedtime and wake-up time recorded in the diary for each period^d^ Bedtime recorded in the diary for each period and bedtime sent from iAide^d^ Steps recorded by iAide2^d^ Achievement rate of target bedtime^e^ Number of barriers and coping strategies set and implemented in the diary for the intervention period^e^ Percentage of use of each drug^f^ Percentage of new additions for each drug since consent was obtained^g^ Changes in antidiabetic drugs since consent was obtained (strengthened, unchanged, or attenuated)^g^

^a^Evaluate the differences after the end of the intervention period (at 12 weeks) and after the end of the observation period (at 24 weeks) from the time of consent acquisition.

^b^PSQI-J: Pittsburgh Sleep Quality Index, Japanese version.

^c^Evaluate the differences in the measured values for the following items at 10 weeks and after the end of the observation period (24 weeks).

^d^Evaluate the differences in the average change from the trial period in the 2 weeks before the end of the intervention period.

^e^Evaluate the rates weekly during the intervention period, for the intervention group.

^f^Evaluate the differences in percentages at the time of consent acquisition, at the time of actual registration, after the end of the intervention period, and after the end of the observation period.

^g^Evaluate the difference in proportions after the intervention period and after the observation period.

### Statistical Analysis

In this study, we define 3 analysis populations: the full analysis set (FAS), the per-protocol set (PPS), and the safety analysis set (SAS). The FAS includes all patients for whom the primary outcome was obtained. Following the intention-to-treat principle, we will analyze data based on the assigned group in the FAS analysis. The PPS consists of the FAS population, excluding patients who violated eligibility criteria after randomization, had 7 or more contiguous days of nonuse of the iAide2, or took medications affecting sleep. The SAS includes all patients who used the iAide2 at least once after randomization. In the safety analysis using the SAS, we will analyze data based on the intervention actually used by patients, regardless of allocation. The FAS will serve as the primary analysis population, while the PPS will provide supportive results. All safety analyses will be conducted using the SAS.

Patient characteristics will be presented as mean, SD, minimum, 25th percentile, median, 75th percentile, and maximum for continuous variables, and as frequency and proportion for categorical variables.

In the primary analysis, we will compare the change in HbA_1c_ from baseline (E0) to week 12 (E3; or the point of discontinuation, if earlier) between groups using analysis of covariance, with baseline HbA_1c_ (ie, HbA_1c_ at E0) included as a covariate in the FAS. For participants who discontinue the study, the most recent HbA_1c_ measurement before E3 will be used.

We will also conduct 2 subgroup analyses: one based on baseline HbA_1c_ at E0 (<8.5% or ≥8.5%) and the other based on age at initial registration (E0; <65 or ≥65 years). Subgroup analyses of the primary outcome will be performed in each analysis set to assess whether the efficacy of BedTime is consistent across these subgroups.

In the secondary analysis, changes in HbA_1c_ at week 12 (E3) and week 24 (E4) will be analyzed using the same approach as in the primary analysis. The proportion of patients with HbA_1c_ below 7% at E3 and E4 (or at the point of discontinuation, if earlier) will be compared between groups using the Fisher exact test. Changes from baseline in step counts, various laboratory test values, and questionnaire scores at E3 and E4 (or at the point of discontinuation, if earlier) will also be analyzed as key endpoints. Changes in T2D medications, categorized as weakened, unchanged, or strengthened, will be compared between groups using the Cochran-Mantel-Haenszel test. The proportion of newly added medications will be compared between groups using the Fisher exact test. A paired (2-sample) *t* test will be used for within-group comparisons of changes in TPB Questionnaire measurements over weeks 0 (E1), 10 (E2), 12 (E3), and 24 (E4). Changes in TPB Questionnaire and SRB Questionnaire measurements between week 12 (E3) and week 24 (E4) will be compared using a *t* test. A paired *t* test will also be used for within-group comparisons from week 0 (E1) to week 12 (E3) for goal achievement rate, goal increment rate, goal reduction rate, average number of coping strategies implemented per day, and the number of identified barriers.

As an exploratory analysis, we will use linear regression to examine the relationship between the average number of coping strategies implemented per day and improvement in HbA_1c_ within the intervention group. The model will include the average number of coping strategies implemented per day and baseline HbA_1c_ as explanatory variables, with the change in HbA_1c_ as the response variable. Changes from baseline to week 12 (E3) and week 24 (E4) (or the point of discontinuation, if earlier) will be assessed.

We will perform a statistical analysis of safety outcomes by calculating the frequency, number of cases, and percentage of individuals experiencing adverse events during the trial, intervention, and observation periods. For the intervention and observation periods, data will be compiled separately for each group, and the percentages will be compared between groups using the Fisher exact test.

## Results

**Figure 4:**
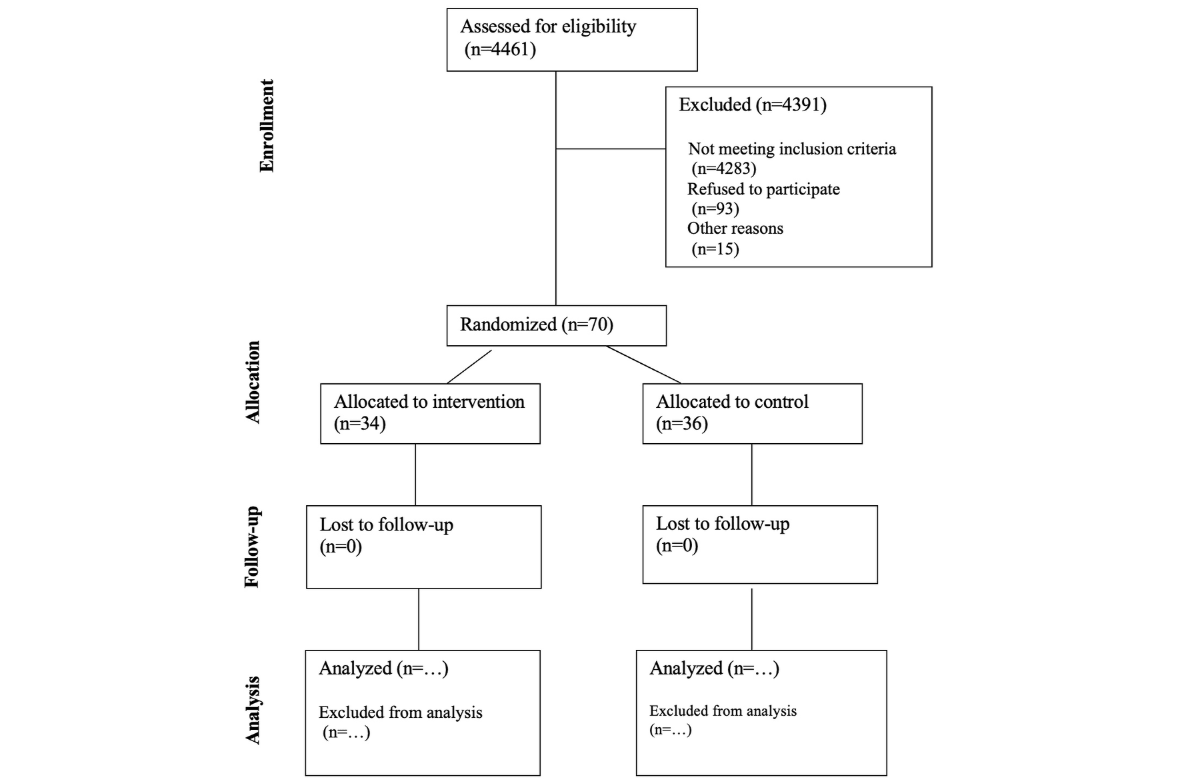
CONSORT (Consolidated Standards of Reporting Trails) flow diagram depicting enrollment, allocation, follow-up, and analysis phases.

Recruitment began on February 8, 2024, and ended on August 29, 2024. The intervention period concluded on December 6, 2024, and the observation period ended on February 26, 2025, with 70 participants completing the observation period (Figure 4). The analysis is currently underway, and we anticipate publishing the results in July 2025. Before commencing analysis, we will conduct a thorough review of all collected data and data entry.

## Discussion

We designed this study based on the foundational concept that it is a behavioral change intervention, despite measuring medical outcomes. The intervention is grounded in a specific theory of behavioral change (TPB) to provide a structured framework, ensure clarity on intervention targets, and maintain focus, avoiding a scattershot approach. We identified a specific, actionable target behavior—going to bed earlier—and developed a measurement and feedback system centered on this behavior. This focused approach provides clarity for both researchers and patients. By maintaining this focus, we can determine whether the intervention effectively changes sleep behavior while also evaluating its impact on sleep patterns and, ultimately, health outcomes. Demonstrating that a behavioral intervention improves HbA_1c_ is not enough, we need to understand the intermediate steps, particularly the behavioral response, to refine the intervention by enhancing effective components and eliminating ineffective ones. This study is designed to generate evidence on these intermediate stages.

Our study utilizes objectively measured data for all primary outcomes and many secondary outcomes. The intervention leverages wireless technology, enabling automatic synchronization of sleep and step count data to the server. These simple, passive features facilitate easy-to-use objective measurements, particularly for older patients.

Participants will be blinded to group assignments to minimize social desirability bias. To achieve this, they will not be informed that the study involves random assignment to 2 groups or that the intervention specifically targets advancing bedtime. Instead, patients will be told that the study examines the relationship between self-management of lifestyle habits (including exercise and sleep) and blood glucose control in individuals with T2D. They will also be informed that various lifestyle factors, including sleep, will be monitored. This approach minimizes the risk of unblinding in the control group by preventing participants from identifying the absence of an intervention. We believe that maintaining blinding offers significant scientific benefits by reducing potential bias and enhancing the validity of the study findings.

We selected the HbA_1c_ level as our primary outcome, as it is recognized as a global standard for assessing glycemic control and an appropriate indicator of T2D treatment effectiveness [77]. According to the Japan Diabetes Society’s treatment guidelines, patients with T2D should aim to lower their HbA_1c_ level below 7% to prevent complications [78]. Therefore, in addition to analyzing absolute changes in HbA_1c_ levels, we examined the proportion of patients achieving an HbA_1c_ level below 7%. We acknowledge that T2D duration may influence the primary outcome. Although our recruitment process does not explicitly stratify participants by disease duration, this information is collected at registration and will be incorporated into the analysis to account for its potential impact on study outcomes.

In this study, we have chosen a “formidable” control [79], using the same devices as the intervention group. As an initial proof-of-concept study, our goal is to determine whether our specific behavior change intervention is more effective than simply having patients record their bedtime and risetime. Evidence suggests that providing patients with a pedometer can increase their activity levels [80], raising concerns that bedtime and risetime recording in the control group may similarly trigger behavioral changes, potentially making it more challenging to demonstrate the intervention’s superiority statistically. By providing the control group with a diary but withholding the intervention functionality, we have designed the study to offer strong evidence of the intervention’s effectiveness—should it indeed be effective.

Many behavior interventions demonstrate solid short-term gains that later fade [39]. This study includes a 12-week observation period following the intervention to assess whether the benefits persist or diminish. Behavior change maintenance is theorized to result from active, ongoing self-regulation, with habit formation emerging after a period of successfully regulating a new behavior [81]. Our goal is to foster sustainable behavior change, ensuring that the new pattern becomes an enduring habit.

A key aspect of our approach is providing patients with a strong introduction to the mechanics of the intervention. Given their busy lives, we strive to make participation clear and manageable. By offering thorough training and ensuring that all intervention messaging remains simple and focused, we maximize the likelihood that patients will engage with the tools as intended.

Another key aspect of our approach is prioritizing the needs of the patients the intervention is designed to serve. Rather than adopting a one-size-fits-all approach, we tailored the intervention for a specific target population, ensuring it aligns with their needs and preferences. Our previous study [39] highlighted the importance of considering cultural background and patient preferences in intervention design. Additionally, we recognize that socioeconomic background may influence sleep patterns. While our recruitment strategy does not explicitly target specific socioeconomic groups, the participating institutions serve patient populations whose demographics are representative of the general community.

Another key aspect of our approach is applying the intervention only to patients who are already motivated to change their behavior. We use the TTM stage as a filter, an approach that has proven highly effective in previous research [39,63]. Our intervention is specifically designed for highly motivated patients who need support in implementing their desired behavior changes, whereas low-motivation patients require a different intervention focused on enhancing motivation. Notably, a large proportion of patients with T2D in Japan exhibit high motivation, with one study indicating that 92% are in the contemplation-through-action stages of TTM for dietary behavior change [82]. There is a substantial pool of patients who could benefit from this targeted intervention. As a multicenter randomized controlled trial, this study enhances the generalizability of our findings to patients with T2D across Japan. Moreover, our techniques are broadly applicable, and there is strong reason to believe that these methods could be adapted to increase sleep duration for patients with other conditions and in diverse health care settings worldwide.

This study has several limitations. The findings may be specific to the population studied and may not fully generalize to other groups. Our participants are likely to be older individuals of Japanese ethnicity, and differences in lifestyle and T2D pathophysiology between Japanese and other populations may limit broader applicability. Additionally, the study includes only patients capable of using mobile phones, introducing potential biases related to digital literacy. Furthermore, our baseline sleep measurements rely on actigraphy and sleep diaries provided to participants, which may overestimate true prestudy sleep levels. This effect is mitigated by the 2-arm design and the duration of our trial. However, it may lead to an underestimation of increases in sleep duration, potentially resulting in an overestimation of the per-hour-of-sleep impact on health outcomes.

This trial will provide important evidence on the efficacy of a TPB-based mHealth intervention in improving sleep duration and glycemic control in patients with T2D. If the intervention is proven effective and safe, this study could serve as a key step toward integrating mHealth into standard T2D treatment in Japan.

## Abbreviations

ABC: actual behavioral control
CONSORT: Consolidated Standards of Reporting Trails
CRC: clinical research coordinator
FAS: full analysis set
HbA_1c_: hemoglobin A_1c_
mHealth: mobile health
PBC: perceived behavioral control
PPS: per-protocol set
SAS: safety analysis set
SRB: Sleep-Related Behavior
T2D: type 2 diabetes mellitus
TIB: time in bed
TPB: Theory of Planned Behavior
TTM: Transtheoretical Model

## Data Availability

The data sets generated during or analyzed during this study can be obtained from the corresponding author on reasonable request.
